# Notes and Comments

**Published:** 1898-03

**Authors:** 


### Notes and Comments.[1]

NATURE AS AN EDUCATOR.—Dr. Holbrook, in the *Scientific American*, says that nature is, in the physical sense, the father and mother of us all, and that a child that grows up to maturity with a genuine love of the rocks and trees, flowers and insects, storm and sunshine, fresh air and the ocean wave, and all the activities about us, stands a better chance of possessing a healthy nervous system and of maintaining it during life. Nature is a great book, whose authorship is the infinite, and I am not in sympathy with any system of education which takes children, young or old, far away from nature.

TREATMENT FOR CARBOLIC ACID BURNS.—Bathe the burned tissue freely with alcohol, or touch the burned area with chloroform. If this is not employed soon after the burn, however, it will have little or no effect. The *University Magazine* says, when some time has elapsed since the burning, brush the parts with a saturated solution of picric acid in water.

THE TRANS-MISSISSIPPI EXPOSITION, in 1898, promises to be a wonder. An immense fund has been raised for its success. The National Dental Association members need have no fear that Omaha cannot accommodate all who attend. Similar doubts were expressed when the American Association decided to go to Excel-

[1] The assistant editor solicits contributions for this department,—new methods, new remedies and formulas, or any short practical note which may prove of value to the practitioner or student. Address 1718 Walnut Street, Philadelphia.

sior Springs, near Kansas City, a few years ago, but the result was a successful gathering and perfect satisfaction with the accommodations. The Western meetings at Minneapolis and Excelsior were among the best in the history of the American Association.—*Western Dental Journal.*

---

Burned by Röntgen Rays.—A young woman who had the X-rays used recently to determine the nature of a supposed affection of the antrum of Highmore has since lost all the hair from the side of the head exposed to the rays, and the skin of her face and neck were also blistered. This is of the same nature as a case reported by the writer over a year ago, where a young man had the right cheek exposed to the rays several times for about five minutes at each sitting. The result was that in a few days he lost all of his beard on that side of his face.

---

Novel Method of Arresting Hemorrhage.—Dr. Bienwald describes, in the *Semaine Médicale*, an ingenious method employed by him to control the bleeding from a small wound of the face in a case of hæmophilia in a child two years old. Having failed to arrest the hemorrhage by the application of ferric chloride, some blood was obtained by aspiration from a healthy subject and deposited upon the wound. In a few minutes coagulation took place and the hemorrhage at once ceased.—*Dental Digest.*

---

The Blue Pencil.—The following suggestions are given by Dr. Barrett in the *Dental Practitioner:* A blue or red aniline pencil is a great convenience to the dentist, in either the laboratory or operating-room. It serves to mark a cast for relief, or for the borders of the plates, the positions of the teeth, etc. But its greatest usefulness is found in articulating and grinding artificial teeth, crowns, fillings, etc. The surface to which a crown or filling is to be adapted is heavily marked with the pencil, and the occlusion then made. The pencil mark is transferred to the exact point to be ground off. It is quite as effective and much more convenient of application than the articulating paper sold for that purpose. Keep an aniline pencil constantly within reach when at your work, and it is surprising how often it will be found useful.

Adhesive Cement for Plate- and Bridge-Work.—It is always desirable to have on hand a reliable cement for repairing plaster impressions, for the temporary adjustment of clasps upon plates, for the "waxing up" of teeth in plate- or crown-work, etc., and the following formula is presented for that purpose by Professor W. F. Litch:

Take of

| | |
|---|---|
| Pitch | 100 grains. |
| Gutta-percha | 100 grains. |
| Gum dammar | 150 grains. |

*Process.*—Melt the pitch in an iron pot over a sand-bath; cut the gutta-percha into small pieces and add piece by piece, stirring constantly until all are thoroughly dissolved, then add the gum dammar, and continue stirring until it is melted and completely mixed with the other ingredients.

Finally pour the melted mass into a large basin of cold water, and before it has entirely hardened remove and cut into strips of suitable size.

---

Foreign Restrictive Legislation.—Recent legislation in France makes it practically impossible for a foreigner to there practise medicine. Before being permitted to practise, regardless of his previous qualification, he is required to first acquire the French B.A. degree, and then go through a five years' curriculum. This means that he must spend seven years in France before being allowed to practise, and this, no matter who he may be, how well known, or what position he may have occupied.—*New York Medical Journal*, vol. lxvi., November 13, 1897, page 674; from the *Boston Medical and Surgical Journal*, October 28, 1897.

---

In making gold solder, pure zinc should always be used, the chemically pure metal prepared for the use of analytical chemists. It is desirable, also, to use for this purpose pure metals only, and especially for bridge-work to strictly adhere to a fixed formula. Toilet-pins, brass, spelter, brass wire, etc., are all of unknown composition. We may know the principal metals of which they are composed, but of the impurities always found in metals, as commercially sold and used, we know and can know but little. When pure metals only are used, gold solder can be made as tough and free from brittleness as good gold plate.

